# Pure platelet-rich plasma facilitates the repair of damaged cartilage and synovium in a rabbit hemorrhagic arthritis knee model

**DOI:** 10.1186/s13075-020-02155-6

**Published:** 2020-04-05

**Authors:** Yulun Xue, Xinlin Su, Miao Jiang, Ziqiang Yu, Huilin Yang, Ling Qin, Peter V. Giannoudis, Jiong Jiong Guo

**Affiliations:** 1 Department of Orthopedics, The First Affiliated Hospital of Soochow University, 188 Shizi Street, Suzhou, 215006 People’s Republic of China; 2 Department of Hematology, National Clinical Research Center for Hematologic Disease of PR China, The First Affiliated Hospital of Soochow University, Suzhou, People’s Republic of China; 3 Jiangsu Institute of Hematology, Key Laboratory of Thrombosis and Hemostasis of Ministry of Health of PR China, Suzhou, People’s Republic of China; 4 Department of Orthopaedics & Traumatology, The Chinese University of Hong Kong, Hong Kong SAR, People’s Republic of China; 5 Leeds Orthopaedic Trauma Sciences, Leeds Institute of Rheumatic and Musculoskeletal Medicine (LIRMM), Leeds University, Leeds, UK

**Keywords:** Hemorrhagic arthritis, P-PRP, Synovium, Cartilage

## Abstract

**Objective:**

Hemorrhagic arthritis (HA) is a common disease of the musculoskeletal system caused by hemorrhage in the joints, leading to damages in the synovium and cartilage. Pure platelet-rich plasma (P-PRP) has been recently demonstrated to have anti-inflammatory and regenerative potential attributed to the various cytokines and growth factors that it contains. The aim of this study was to investigate the efficacy of P-PRP for the treatment of patients with mild and severe HA.

**Methods:**

Autologous blood was withdrawn from the New Zealand rabbits and injected into their left and right knees to establish the HA models. The injection was performed once a week and repeated for 8 weeks to establish the mild HA models and for 16 weeks to establish the severe HA models. One hundred microliters of P-PRP was injected into the left HA knees, and the same volume of sterile 0.9% saline was injected into the corresponding right knees. MRI examination, H&E staining, and toluidine blue staining were respectively performed to evaluate the histological difference of synovium and cartilage between the P-PRP treated and untreated mild and severe groups. Normal knees were set as the control group.

**Results:**

Pathological changes including tissue color, joint effusion, and synovium inflammation in the mild treated group were less severe compared to the other three experimental groups based on gross observation. The difference of joint cavity diameter between the mild treated and untreated groups was 2.67 ± 0.75 mm (95%CI, 1.20–4.14 mm), which was significantly larger than that between the severe treated and untreated groups (1.50 mm ± 0.48, 95%CI, 0.56–2.44 mm) (mean difference in joint cavity, 1.17 ± 0.32 mm; 95%CI, 0.49–1.85 mm; *P* < 0.01). MRI examination showed the synovitis and bone marrow edema were less severe in the treated groups compared to the corresponding untreated groups. H&E staining of the synovium suggested that the inflammation was much less and the cell number was much smaller in the treated than in the untreated HA knees in regard to both the mild and severe groups. The mean difference of cell number between the mild treated and untreated groups was 307.40 ± 14.23 (95%CI, 241.54–343.26; *P* < 0.001), which was 699.20 ± 82.80 (95%CI, 508.26–890.14; *P* < 0.001) between the severe treated and untreated groups. H&E staining and toluidine blue staining of the cartilage exhibited an obvious amelioration of inflammation and cartilage matrix loss after being treated with P-PRP for both groups, especially the mild group.

**Conclusions:**

P-PRP was effective for the treatment of HA by inhibiting the development of synovitis and cartilage matrix loss in the affected joints, which was particularly obvious in the early-stage HA. This study supports the view that there is a great potential of P-PRP to be considered and used as a non-operative treatment for hemorrhagic arthritis at its early stage.

## Introduction

Hemorrhagic arthritis (HA) is a common articular disorder caused by episodic or persistent bleeding in the joints, leading to damages in affected synovium and cartilage. Accumulation of blood degradation products could instigate inflammatory reactions, resulting in hypertrophy of the synovium and chronic inflammation in the joints. Conservative treatments such as immobilization, analgesia, and anti-inflammatory strategies could ameliorate the symptoms of HA but cannot prevent its progression efficiently [1, 2]. Patients with late-stage HA usually suffer from severe pain, joint deformation, and dysfunction and have to undergo total knee arthroplasty (TKA) in the end. However, since a large proportion of the patients develop HA at a relative young age, TKA is a suboptimal option due to the possibility of revision surgeries in the future [3].

As an autologous blood derived product, platelet-rich plasma (PRP) contains a large amount of platelets and diverse growth factors, which in recent years has been increasingly demonstrated to be effective for the treatment of many diseases [4–6]. Caviglia et al. reported that PRP was effective and safe for the treatment of chronic hemophilic synovitis [7]. Moussa et al. suggested that PRP could be a potential therapy for the treatment of osteoarthritis (OA) [8]. The therapy potential of PRP is attributed to various cytokines and growth factors, such as platelet-derived growth factors (PDGF), transforming growth factors β1 (TGFβ1), insulin-like growth factors (IGF), platelet factor 4 (PF-4), fibroblast growth factor 2 (FGF-2), and vascular endothelial growth factor (VEGF), which could stimulate the proliferation ability of tissue-specific cells, increase the production of functional proteins and anti-inflammatory molecules, and eventually accelerate the healing process [9, 10].

Interestingly, it has been reported that the constitution and concentration of components in PRP varied due to different preparation methods and apparatus, which might influence the treatment outcome [11, 12]. Based on the abundance of leukocytes, the PRP can be classified into pure PRP with a low concentration of leukocytes (P-PRP) and PRP with a high concentration of leukocytes (L-PRP). Leukocytes on one hand have the antimicrobial and immune regulation potential physiologically; on the other hand, they can release inflammatory cytokines such as IL-1, IL-6, and TNF-α under certain circumstances, which might promote inflammation in target tissues and affect the healing process. A recent study conducted by Cavallo et al. [3] demonstrated that P-PRP could induce the production of diverse extracellular matrix molecules more efficiently compared to L-PRP. It is thus assumed that P-PRP is more effective in treating patients with aseptic injuries. According to our knowledge, few publications have reported the therapeutic effect of P-PRP on patients with traumatic or pathological hemorrhagic arthritis [12]. The aim of this study therefore was to investigate the therapeutic effect of P-PRP in treating mild and severe HA by developing corresponding rabbit HA models, which could help explore more effective strategies for treating patients with HA.

## Methods

### Establishment of the knee HA models using New Zealand rabbits

**Fig. 1:**
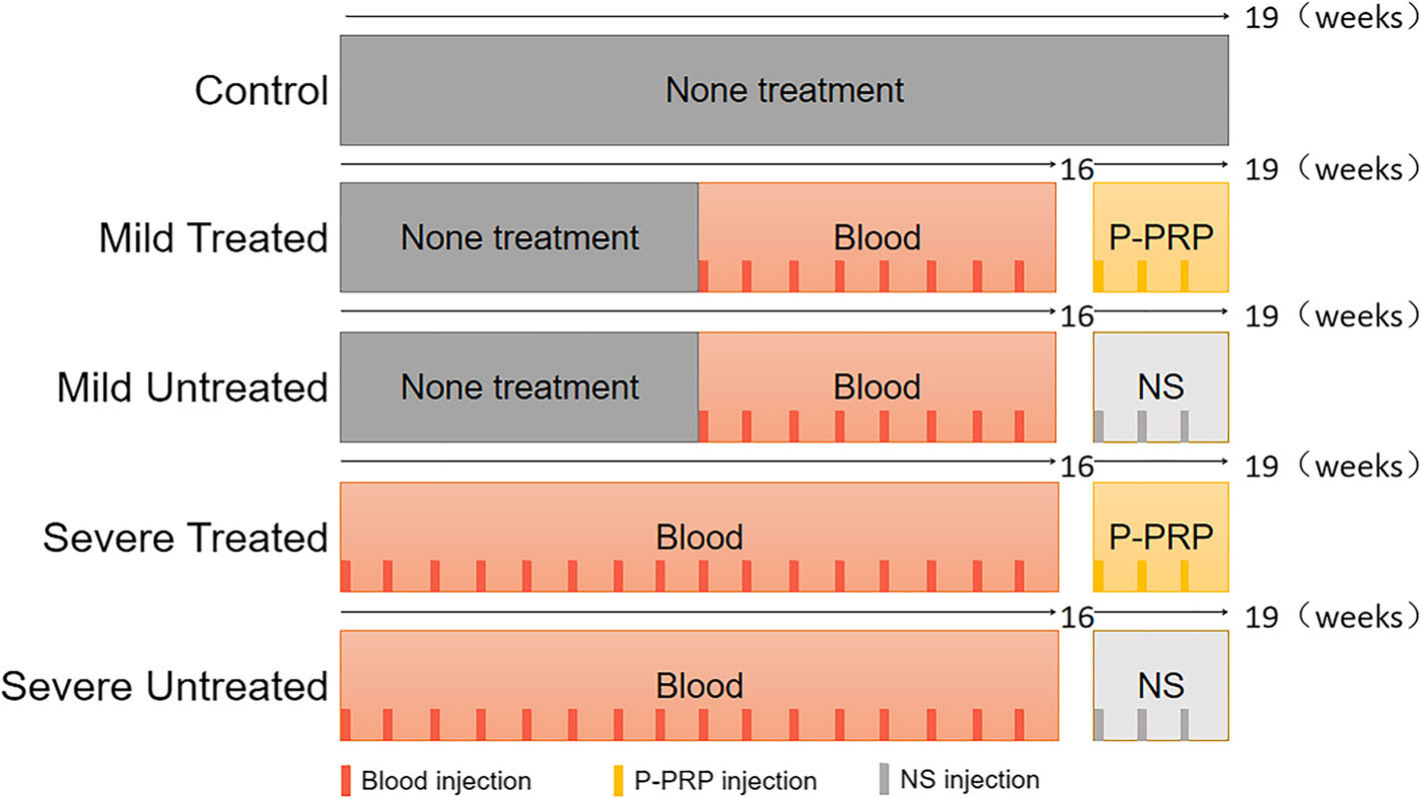
Grouping situation of the New Zealand rabbits. P-PRP, pure platelet-rich plasma; NS, normal saline; Blood, autologous whole blood. Control group (*n* = 4), none treatment; mild groups (*n* = 8), left knee was the mild treated group and right knee was the mild untreated group; severe groups (*n* = 8), left knee was the severe treated group and right knee was the severe untreated group

This study was approved by the ethical committee of the animal center of the Medical College of Soochow University. According to Shammas et al. and Wang et al. [13, 14], 20 male SPF New Zealand rabbits (10 weeks of age) weighing 2.5–3.0 kg were used to establish the rabbit knee HA models. They were firstly anesthetized by 10% chloral hydrate solution. Two milliliters of blood was drawn from the ear vein of each rabbit and injected into the knee joints. If the blood was coagulated before injection, the blood drawing would be repeated. Blood injection was performed once a week. Rabbits (*n* = 8) in the mild group were injected for 8 weeks whereas those (*n* = 8) in the severe group were injected for 16 weeks. The severe groups (*n* = 8) were firstly injected for 8 weeks, and then together with the mild groups underwent another 8 week of blood injection [13–15]. Therefore, rabbits in two groups were at the same age when processed for P-PRP treatment. Four normal healthy male rabbits at the same age were set as the control group. The method of grouping and design of this study were summarized in Fig. 1.

### Preparation of P-PRP

P-PRP was prepared according to Dohan Ehrenfest et al. and Franco et al.’s studies [16, 17]. The rabbits were anesthetized by 10% chloral hydrate solution. Five milliliters of blood was drawn from the ear artery of each rabbit and then injected into a centrifuge tube containing 1 ml of 4% sodium citrate solution. The tubes were centrifuged under 200×*g* for 10 min. The supernatant was then drawn into a new tube. This step was repeated for 3 times. The summation of the supernatant was thereafter centrifuged under 1000×*g* for 15 min. The supernatant platelet-poor plasma (PPP) and the precipitated platelets were respectively collected. P-PRP was finally obtained by blending the precipitated platelets with 100 μl PPP. Components of P-PRP and whole blood of New Zealand rabbits were respectively detected by using a hemocyte analyzer (Beckman coulter, Z2, USA).

### Treatment of HA with P-PRP

According to Chouhan’s method, 3 times of PRP injection was better in inflammation reduction than a single injection [18]. In this study, both knees of the rabbits were shaved under aseptic condition. One hundred microliters of P-PRP was injected into the left knee, and the same volume of saline was injected into the right knee sterilely through the inferior patellar tendon using a 1-ml syringe with a 25-G needle. The injection was conducted once a week and repeated for 3 weeks.

### MRI assessment

Each rabbit was placed in a prone position and processed for MRI detection using the 1.5-T MRI system machine (Siemens Magnetom 1.5 T, Germany) with standard sequences (including T1WI, T2WI, and PDWI). MRI images from the sagittal, horizontal, and coronal views were respectively analyzed and compared among the experimental and control groups.

### Gross morphology of the knee joints

After the rabbits were euthanized, patellar tendons were cut off to expose the synovium and cartilage of the knee joints. The diameter of the joints was defined as the largest length of the femoral condyles and measured using a fine vernier caliper, which was compared among the mild and severe groups with and without P-PRP treatment. Pathological changes including tissue color, joint effusion, and inflammation of the synovium and cartilage were carefully evaluated among the mild and severe HA knees with or without P-PRP treatment.

### Histological assessment

Synovium derived from the affected knees were fixed with 10% paraformaldehyde for 48 h, decalcified with 14% EDTA for 1 week, dehydrated with graded ethanol solutions, embedded in the optimal cutting temperature compound, and finally sectioned with a thickness of 6 μm. The sections were thereafter stained with hematoxylin and eosin (H&E) for histological assessment. According to Krenn V, evaluation of the hyperplasia of synovial stroma and the severity of inflammatory infiltrate should be performed to make the histomorphological grading of the synovium [19]. The detailed definitions are shown in Table 1.

**Table 1:** Detailed scoring regulations of synovium histomorphology

	Cell stroma	Inflammatory infiltrate
1 point	The cellularity is slightly increased	Few situated lymphocytes or plasma cells
2 points	The cellularity is moderately increased; multinucleated cells might occur	Many lymphocytes or plasma cells, sometimes follicle-like aggregates
3 points	The cellularity is greatly increased; multinucleated giant cells, pannus formation, and granulomas might occur	Dense band-like inflammatory infiltrate or numerous large follicle-like aggregates

The number of synovial and inflammatory cells in the synovium was measured using the ImageJ 1.52a (National Institutes of Health, USA). The hue of the color threshold option was set at 150–200 to cover the color of nucleus of the cells. The saturation and the brightness were adjusted to include all the alternative targets. Then, we analyzed the nucleuses through setting size 4 (pixel^2^) to infinity and circularity as 0.8–1.0.

Cartilage samples along with subchondral bone were cut off at the articular surface and processed for similar procedures except that they were decalcified for 1 month and additionally dealt with toluidine blue staining. Cartilage surface integrity, arrangement of the cell population and ECM, and abundance of the matrix were respectively evaluated and compared among different groups.

### Statistical analysis

The data was analyzed using the SPSS 19.0 (SPSS Inc., Chicago, IL) and presented as means ± standard deviations (SD) along with the 95% confidence interval (95%CI). One-way ANOVA or Welch’s ANOVA (when homogeneity of variance is not homogeneous) were then conducted. The cell counting number and diameter of the knee joints were analyzed and compared using the LSD and Games-Howell tests among different groups. *T* test was used to compare the difference of diameter and synovial score between the treated and untreated groups, and the component difference between P-PRP and whole blood. The *P* value < 0.05 was considered statistically different.

## Results

### Components in P-PRP and whole blood

The concentration of platelets in P-PRP was 790.25 ± 30.29 × 10^6^/ml (95% CI, 742.04–838.45 × 10^6^/ml). The concentration of leukocyte in P-PRP was 0.15 ± 0.03 × 10^6^/ml (95% CI, 0.11–0.19 × 10^6^/ml), which was immensely lower than that in the whole blood (95% CI, 5.43–6.28 × 10^6^/ml, *P* < 0.0001) (Table 2).

**Table 2:** Mean concentrations of platelets, red blood cells, and white blood cells in whole blood and pure platelet-rich plasma

	Platelet concentration (mean ± SD; 95%CI) (× 10^6^/ml)	Leukocyte concentration (mean ± SD; 95%CI) (× 10^6^/ml)	Red blood cell concentration (mean ± SD; 95%CI) (× 10^9^/ml)
Whole blood	199.50 ± 19.22; 179.33–219.67	6.00 ± 0.40; 5.58–6.43	4.48 ± 0.54; 3.91–5.04
Pure platelet-rich plasma	790.25 ± 30.29; 742.04–838.45	0.15 ± 0.03; 0.11–0.19	0.06 ± 0.01; 0.05–0.08
p	< 0.0001	< 0.0001	0.0002

### MRI results

**Fig. 2:**
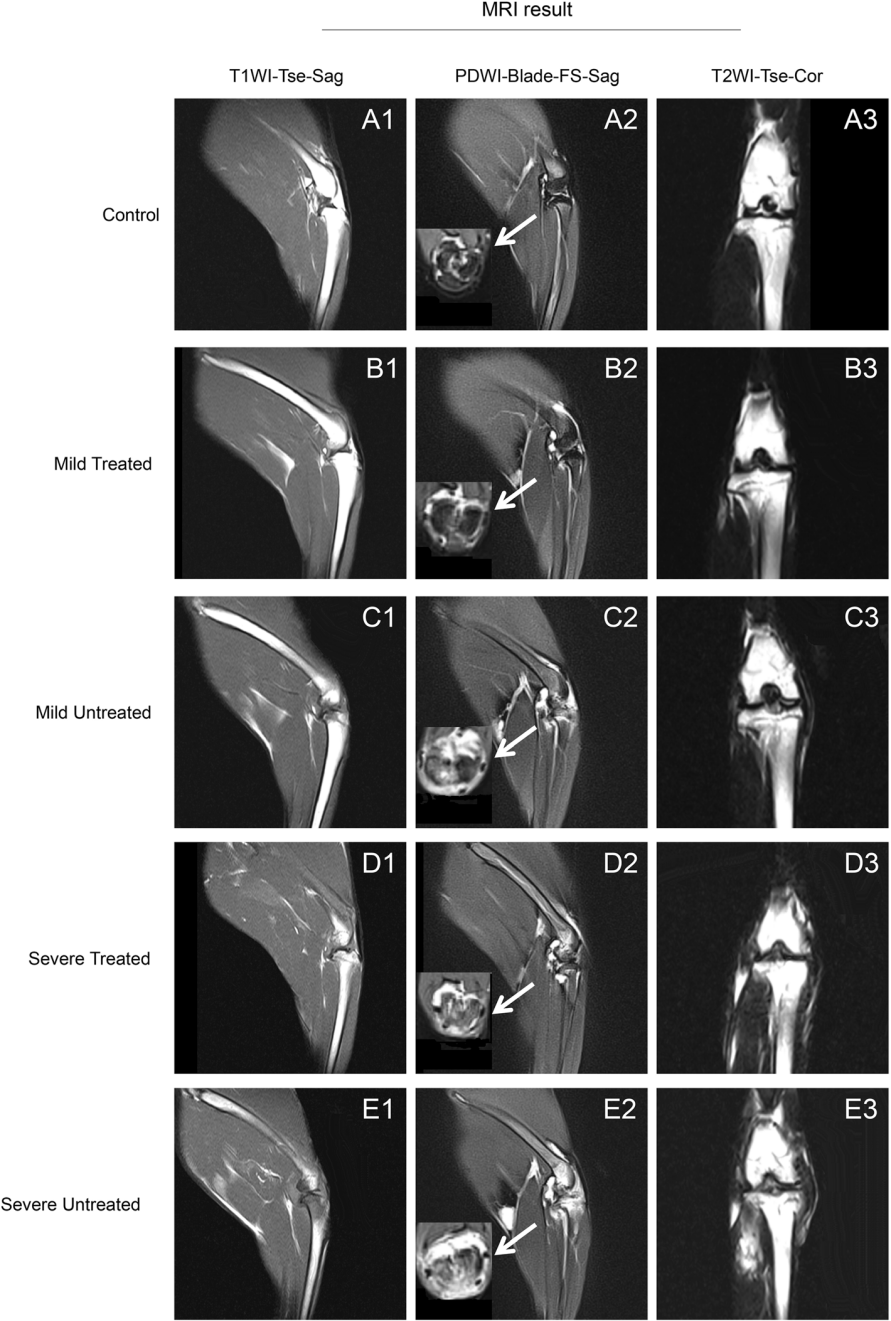
Representative MRI images for imaging diagnosis. T1WI sequence (A1, B1, C1, D1, E1) from the sagittal plane and PDWI-FS (A2, B2, C2, D2, E2) sequence from the sagittal plane and cross plane. T2WI sequence (A3, B3, C3, D3, E3) from the coronal plane

T1WI sequence of MRI could best show the anatomy of normal tissues while T2WI sequence could best show the pathological changes of targeted tissues. It was observed that joint cavity in the severe treated and untreated groups were distinctly enlarged in comparison with their corresponding control groups under the T1WI and T2WI sequences (Fig. 2). The mean joint width of the mild treated group was closest to the control group compared to other experimental groups (Fig. 2). PDWI sequence in sagittal and horizontal views revealed obvious synovitis of knee joints in all experimental groups (Fig. 2). The severe untreated group exhibited the most serious synovitis, cartilage inflammation, and bone marrow edema. The inflammation was apparently less severe in the treated groups than in the untreated groups for both the mild and severe HA knees.

### Gross morphology

**Fig. 3:**
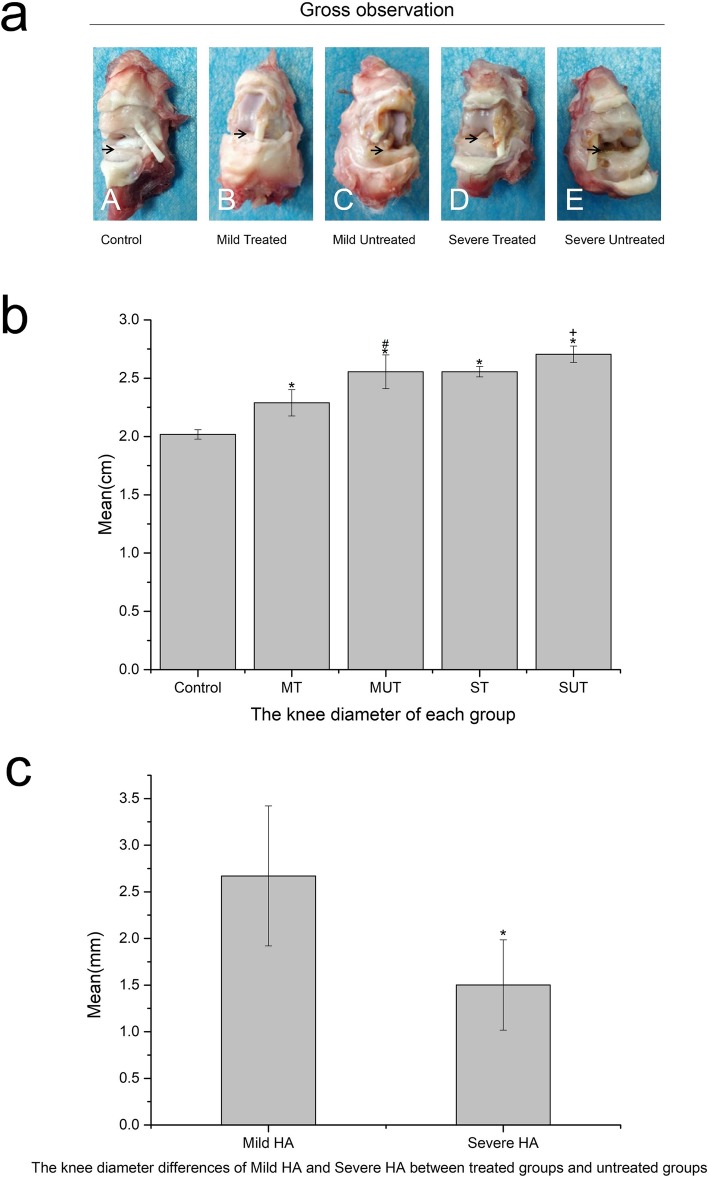
**a** Gross observation of the rabbit knees after the rabbits were euthanized. The tissues around the joint were shown by the arrows. In the control group, the synovium and the surface of the femur condyle were white and smooth. However, in the other four groups, the tissues all turned yellow in different degrees. The mild treated group was most similar to the control group. **b** The diameter of each group. Control, control group; MT, mild treated group; MUT, mild untreated group; ST, severe treated group; SUT, severe untreated group. All four experimental groups had a larger diameter than the control group (*p < 0.05). The diameters of treated groups were smaller than the untreated groups (^#^*p* < 0.05, ^+^*p* < 0.05). Error bars indicated the standard deviations of the mean values. **c** The diameter differences of the knee joint between treated and untreated groups. The mild HA groups (mild treated group and mild untreated group) (2.67 ± 0.75 mm, 95%CI, 1.20–4.14 mm) had a larger diameter difference than the severe HA groups (severe treated group and severe untreated group) (1.50 ± 0.48 mm, 95%CI, 0.56–2.44 mm) (*p < 0.05). Error bars indicated the standard deviations of the mean values

The diameter of the knee joints was measured and compared among the experimental and control groups, which were obviously larger in the experimental groups than in the control group. The diameter of the treated groups was smaller than the corresponding untreated groups. The difference of diameter between the treated and untreated groups was calculated, which revealed that the difference was significantly larger in the mild groups (2.67 ± 0.75 mm, 95%CI, 1.20–4.14 mm) than in the severe groups (1.50 ± 0.48 mm, 95%CI, 0.56–2.44 mm) (mean difference in joint cavity, 1.17 ± 0.32 mm; 95%CI, 0.49–1.85 mm; *P* < 0.01) (Fig. 3, *P < 0.05, ^#^*P* < 0.05, ^+^*P* < 0.05). This result indicated a better therapeutic effect of P-PRP in the mild HA. As shown in Fig. 3, the synovium and articular cartilage of the femur condyle were white and smooth in the control group, which turned yellow and exhibited joint effusion and synovium inflammation in the four experimental groups. These pathological changes in the mild treated group were relatively less severe compared to the other three experimental groups.

### Histological assessment

#### Synovium

**Fig. 4:**
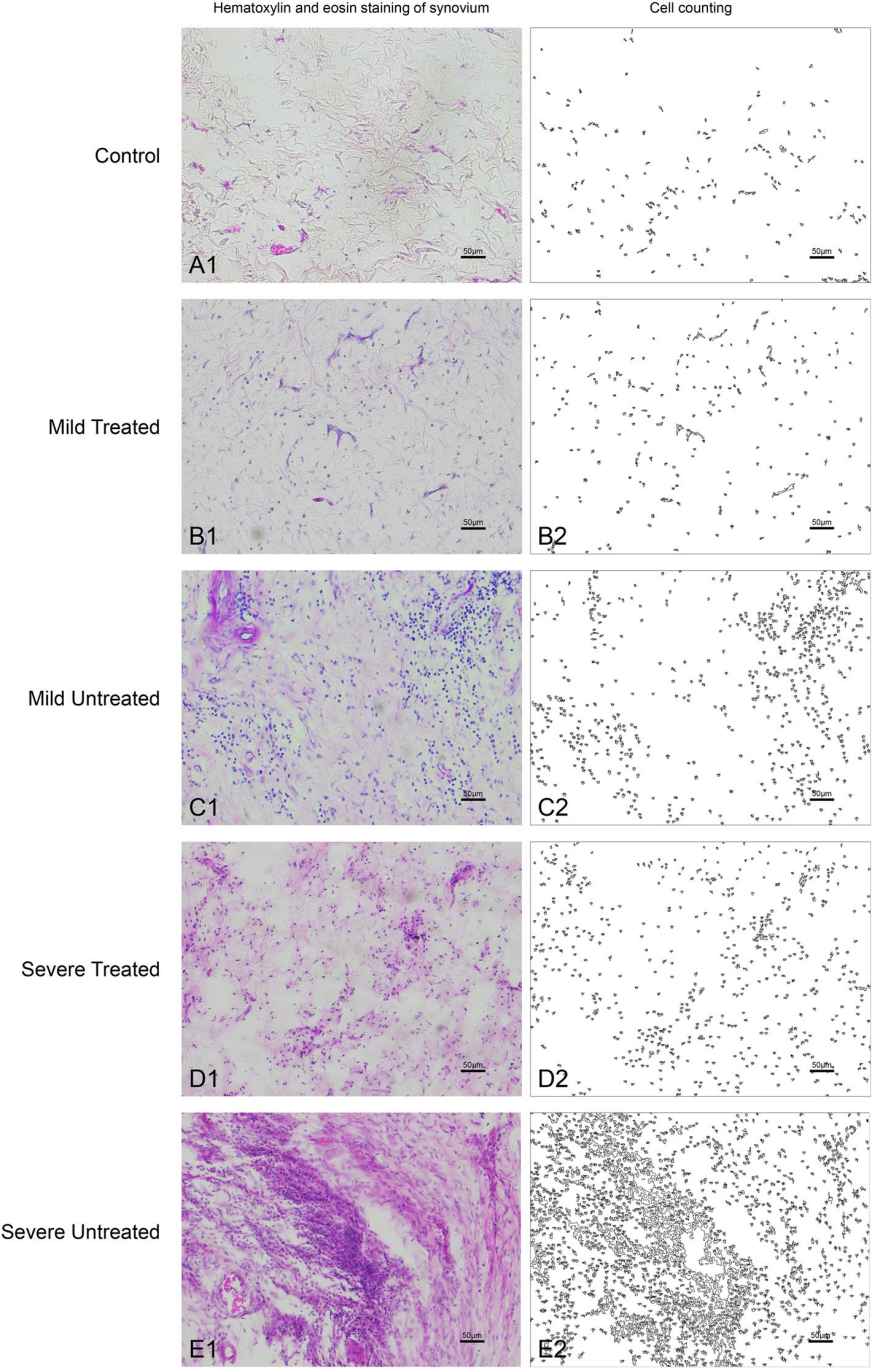
The H&E staining of the synovium in each group and the corresponding processed images for cell counting. Scales represented 100 μm

**Fig. 5:**
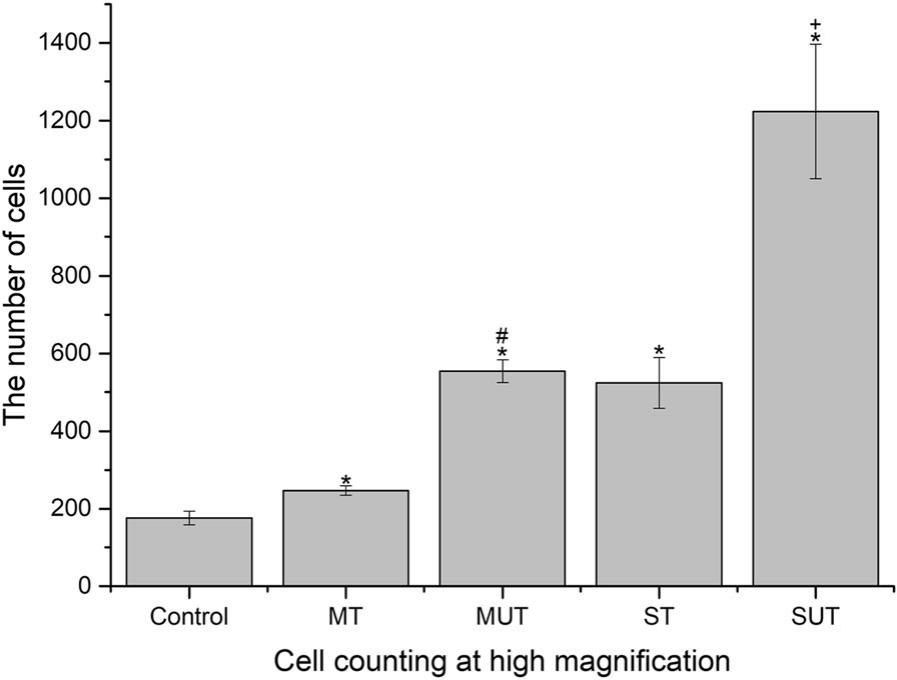
The average cell number of each group (*n* = 8) at high magnification. Control, control group; MT, mild treated group; MUT, mild untreated group; ST, severe treated group; SUT, severe untreated group. *p < 0.05 compared with the control group (*n* = 4). ^#^*p* < 0.05 compared with the mild treated group. ^+^*p* < 0.05 compared with severe treated group. Error bars indicated the standard deviations of the mean values

H&E staining of the synovium showed that the number of vasculatures and inflammatory cells had obviously increased in the HA groups compared to the control group, which were much larger in the severe HA knees than in the mild HA knees and had significantly decreased in both the mild and severe groups after being treated with P-PRP (Fig. 4). The decrease of cell number after treatment was analyzed in the mild and severe groups respectively. It exhibited that the mean decrease of cell number was 307.40 ± 14.23 (95% CI, 241.54–343.26; *P* < 0.001) and 699.20 ± 82.8 (95% CI, 508.26–890.14; *P* < 0.001) for the mild and the severe groups respectively after being treated with P-PRR (Fig. 5).

**Fig. 6:**
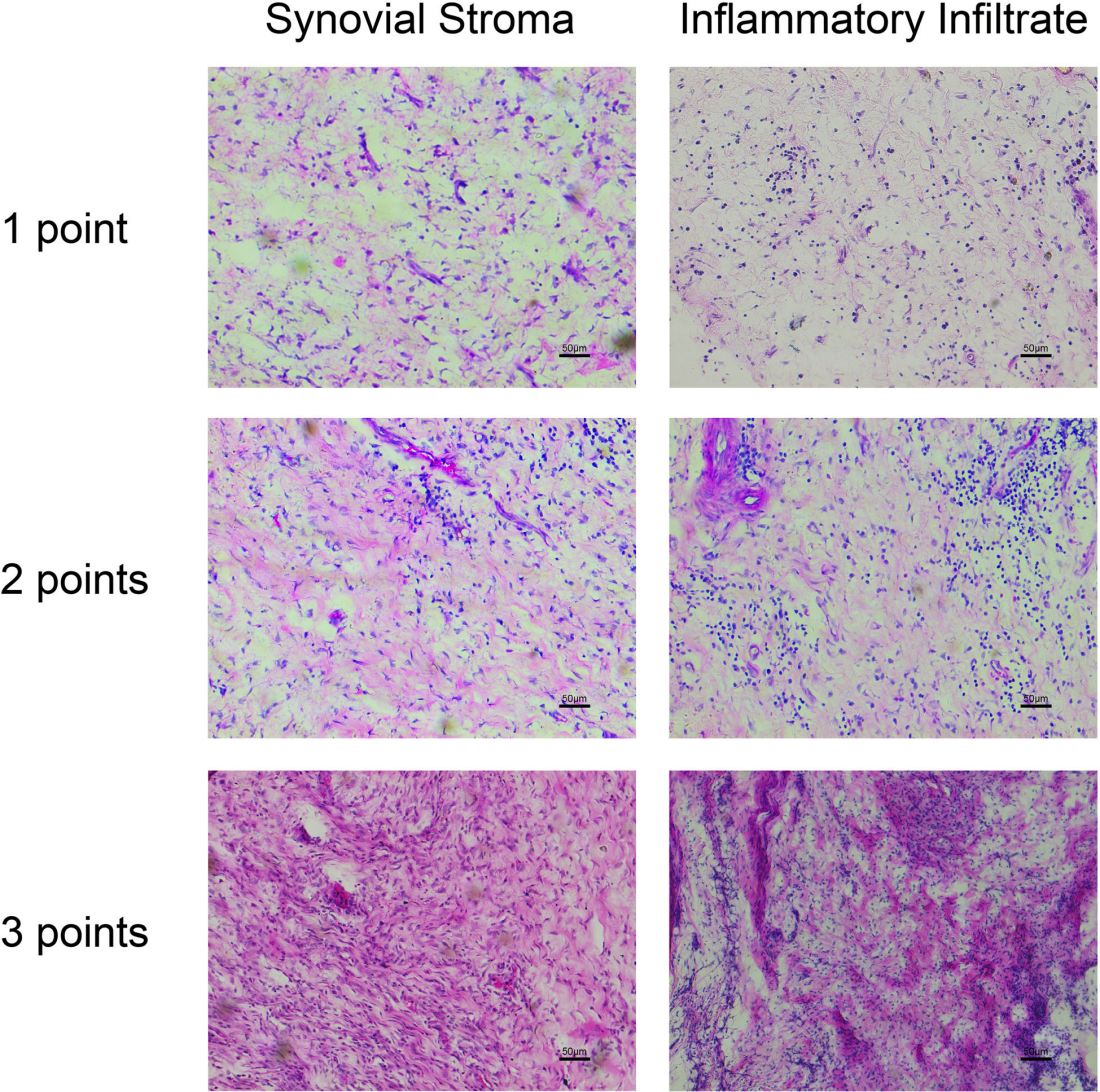
Examples of the synovium histomorphological score of the cellular density of the synovial stroma and the inflammatory infiltrate increasing from top to bottom

**Fig. 7:**
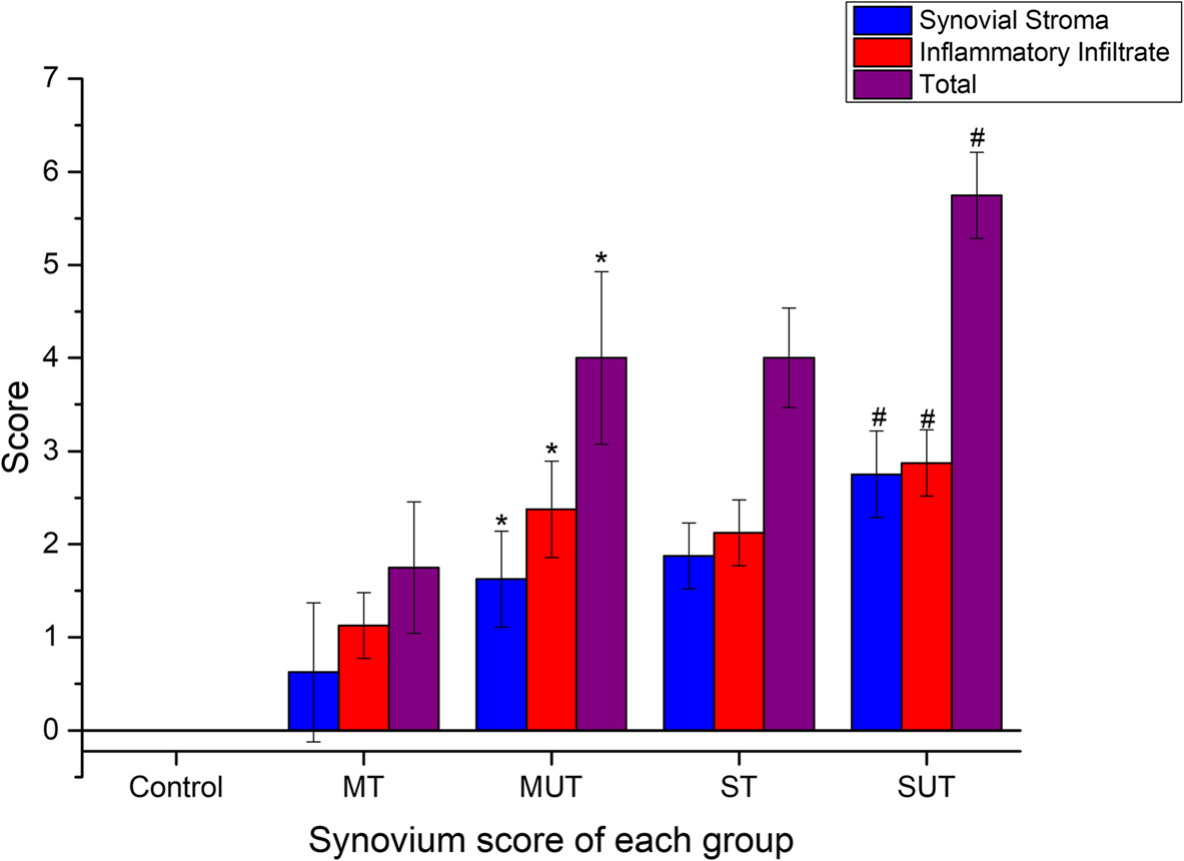
Synovium score of each group. Control, control group; MT, mild treated group; MUT, mild untreated group; ST, severe treated group; SUT, severe untreated group. The three bars respectively represented the synovial stroma score, inflammatory infiltrate score, and the total score from left to right. Error bars indicated the standard deviations of the mean values. *p < 0.05 compared with the MT group. ^#^*p* < 0.05 compared with the ST group

Evaluation of the histomorphological score of the synovium is shown in Fig. 6, which included the hyperplasia of synovial stroma and the inflammatory infiltrate. The former was usually reflected by the increased resident synovial cells and extracellular matrix, while the latter mainly indicated the infiltration of the inflammatory cells, most of which were lymphocytes. Pictures in Fig. 6 exhibited the severity of inflammation in the synovium had increased from the top to the bottom. The histomorphological score of the synovium in each group was evaluated and presented in Fig. 7. The three bars represented the synovial stroma score, inflammatory infiltrate score, and the total score from the left to the right, respectively. It revealed that the score was significantly lower in the treated groups than in the untreated groups for both the mild and severe HA knee (Fig. 7, *P < 0.05, ^#^*P* < 0.05).

**Fig. 8:**
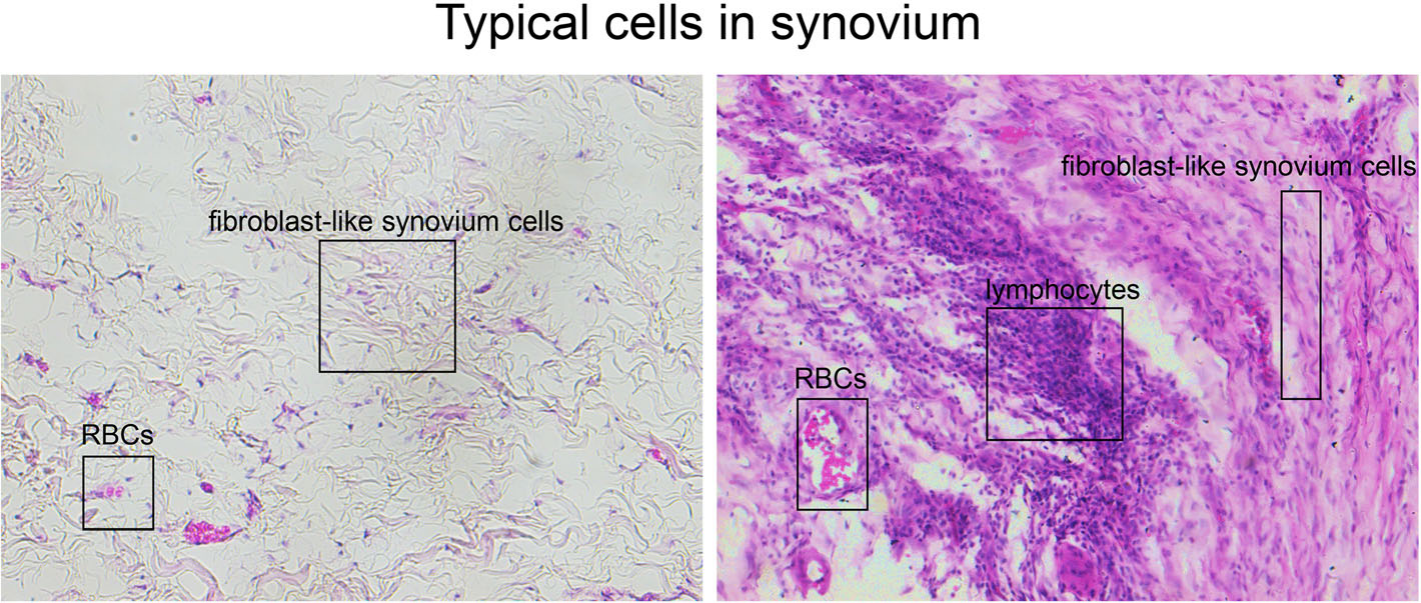
Typical cells of normal and HA in the synovium H&E staining

**Fig. 9:**
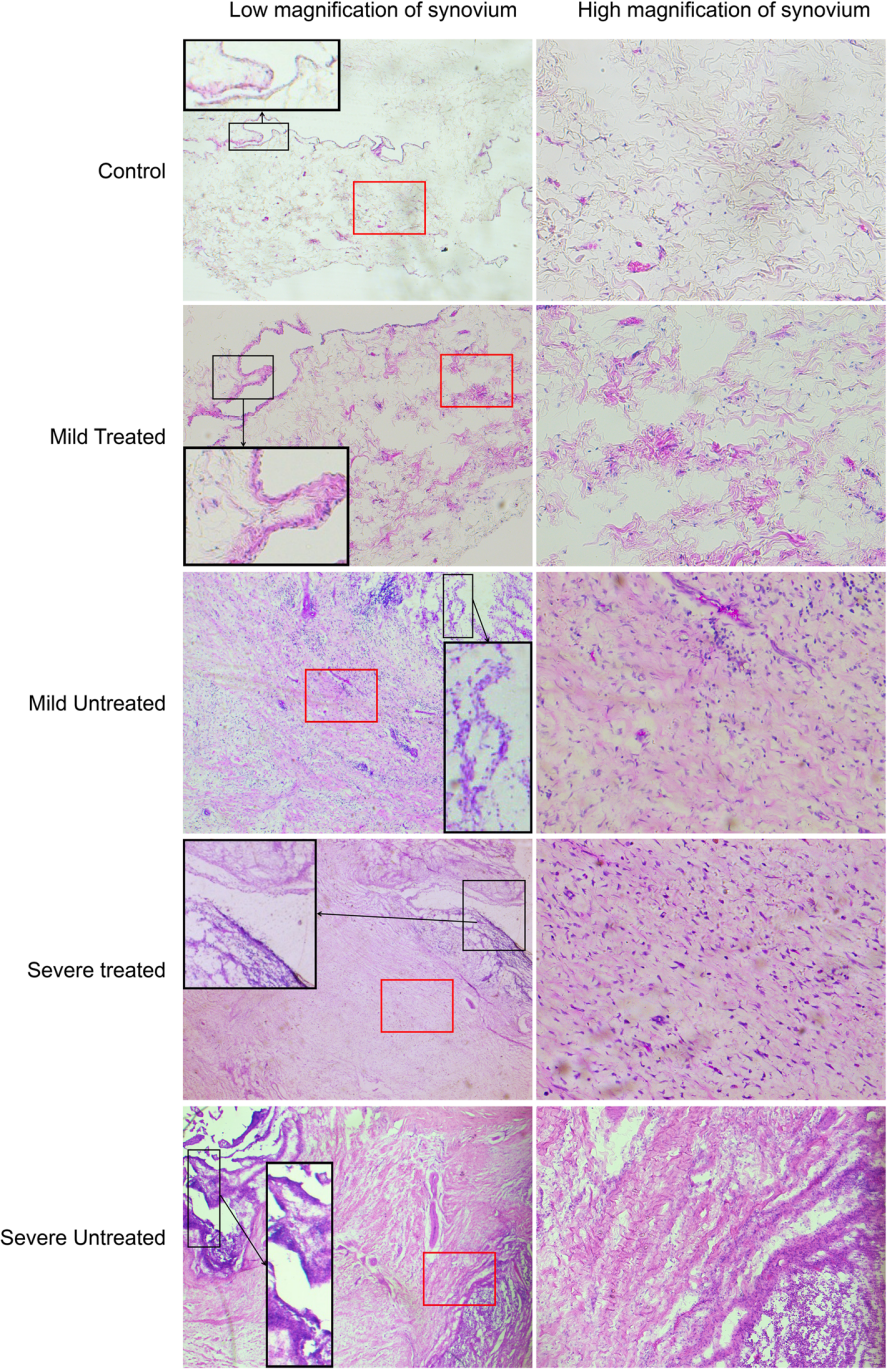
The synovium H&E staining of low and high magnifications. The black frame showed the edge of the synovium turning into palisade-like cells in the experiment groups and gathering band-like lymphocytes in severe HA. The red frame shows the inner side of the synovium with synovial stroma increased and lymphocytes infiltrated

Figure 8 exhibited the vasculatures, resident fibroblast-like synovium cells, and non-resident inflammatory cells in the normal (Fig. 8a) and HA (Fig. 8b) synovium with H&E staining. It was observed that the fibroblast-like synovium cells presented light transplant cytoplasm in the normal synovium, while those in the severe HA synovium presented a much darker staining of cytoplasm. Inflammatory cells, most were lymphocytes, migrated and gathered with a large scale in severe HA. In addition, the amount of vasculatures had also obviously increased in the severe HA synovium. In Fig. 9, the black frame indicated that the edge of the synovium was filled with palisade-like cells and many lymphocytes gathered in experimental groups, especially in severe HA. Compared to the control group, the cell number in the edge layer was obviously increased in the mild untreated group whereas slightly enlarged in the mild treated group. Besides, the number of lymphocytes was especially increased in the severe groups. The red frame indicated the hyperplasia of synovial stroma and infiltration of lymphocytes in the inner region of synovium, which showed that the injury in the treated groups was much less severe than in the untreated groups regarding both mild and severe HA knees.

#### Cartilage

**Fig. 10:**
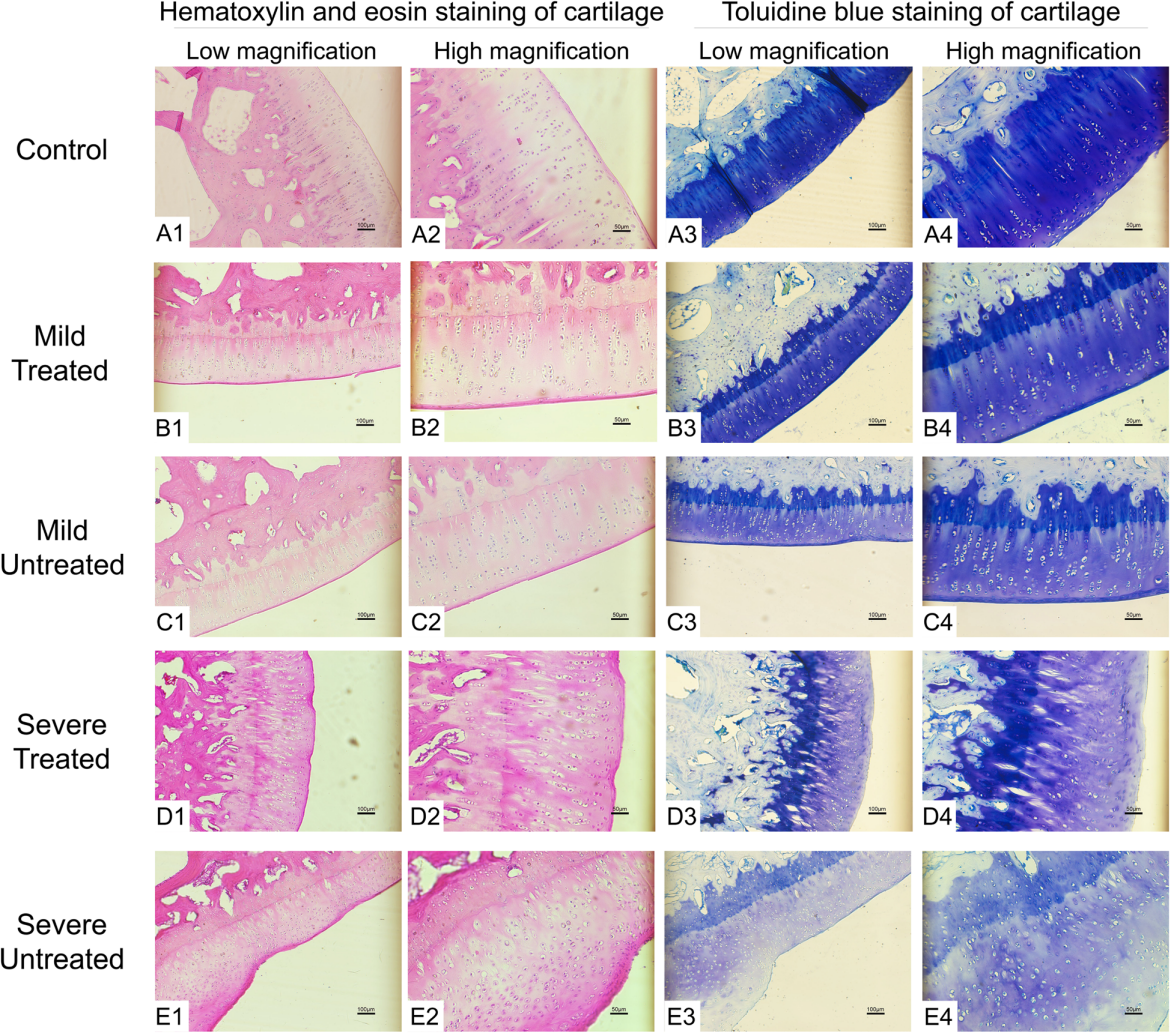
The H&E staining and toluidine blue staining of the cartilage of each group. Scales represented 100 μm at low magnification and 50 μm at high magnification

H&E staining of the cartilage revealed that the number of cartilage cells had increased while the extracellular matrix (ECM) had reduced in the HA groups compared to the control group. However, after being treated with P-PRP, the ECM had relatively increased in both the mild and severe groups (Fig. 10). Toluidine blue staining was further performed to assess the ECM of the affected cartilage. It was observed that the thickness of cartilage and amount of ECM in four HA groups were lower than those in the control group. Compared to the ECM in the mild and severe HA knees without P-PRP treatment, those treated with P-PRP had obviously increased correspondingly. Though the cartilage of the severe untreated group seemed to be increased in the lower left corner of the picture, it could be identified as fibrotic hyperplasia through the disordered cell arrangement and fibrosis on the surface (Fig. 10E1, E3). The cartilage surface was intact and the arrangement of the cell population and the ECM remained in order in the mild treated and untreated groups, whereas in the severe groups, the cartilage surface displayed obvious fibrosis and the cell population and ECM exhibited disordered arrangement, particularly in the severe untreated group (Fig. 10D3, E3). In addition, the subchondral bone in the severe untreated group presents a certain degree of bone loss (Fig. 10E3, E4), which was not observed in the mild groups and the severe treated group. This finding suggested that the subchondral bone could also be affected in the severe HA knee, and P-PRP might be engaged with the potential of promoting the healing process of bone injury.

## Discussion

Patients with HA had constant articular hemorrhage in the affected joints, leading to hypertrophic and inflammatory pathologies of the cartilage and synovium. Containing high-concentration of platelets and various growth factors, P-PRP is presumed to be useful for patients with HA. In the present study, using the rabbit HA model, we compared the pathology of HA with and without P-PRP treatment through MRI and histological detection and found that synovitis became less severe and the number of inflammatory cells and amount of cartilage matrix loss were reduced after P-PRP treatment. We preliminarily demonstrated that P-PRP was effective for HA.

Due to the regenerative and anti-inflammatory properties, PRP was first used in an open heart surgery by Ferrari et al. in 1987 [6]. In recent years, the application of PRP in bone and soft tissue diseases has been increasingly reported, including trauma-related injuries and degenerative diseases [4, 20]. Products combined with PRP and other ingredients like fibrin, adipose tissue, and BMSCs have already been used in fracture, large bone defects, and OA [20–23]. Finding new methods for driving polarization of macrophages may be the next focus on PRP therapies [24]. It has been demonstrated that various growth factors and cytokines play an essential role in the success of PRP therapy. However, concentration of other PRP components like leukocytes could also affect the therapy effect [25, 26]. A standard regimen for the components of PRP and their concentration up to now has not been established. A previous study reported that P-PRP was superior to L-PRP in treating osteoarthritis (OA) using a rabbit OA model, which was developed by anterior cruciate ligament transection [5]. Consequently, we selected P-PRP instead of PRP in our study and validated its efficiency in HA.

Growth factors (GFs) released by platelets are supposed to play an essential role in lowering the inflammation in the synovial tissue, which leads to a reduction in the synovial hyperplasia and cytokines production [7]. The most relevant GFs in PRP are platelet-derived growth factor (PDGF), transforming growth factor beta (TGF-β), fibroblast growth factor (FGF), insulin-like growth factor 1 (IGF-1), connective tissue growth factor (CTGF), epidermal growth factor (EGF), and hepatocyte growth factor (HGF). These GFs also play a key role in regulating and stimulating the healing and regenerative processes of different tissues and are able to induce cell proliferation, matrix remodeling, and angiogenesis [27]. TGF-β is one of the most important GFs that may enhance chondrocyte synthetic activity, matrix production, and cell proliferation and decrease the catabolic activity of IL-1 [12]. An adequate anti-inflammatory response is essential for decreasing tissue damage and increasing tissue repair. In the present study, it was shown that inflammation and damage in both synovium and cartilage was reduced after P-PRP treatment, which suggested that PRP had a regenerative and protective effect for cartilage in addition to the synovium.

The components and their concentration in PRP vary along with different preparation methods depending on the differences in speed and duration of centrifugation. To the best of our knowledge, a standard regimen for the components of PRP has not been established yet. Platelet concentrations between one to nine times higher than whole blood were used in the previous studies in tissue healing and three to four times higher than the baseline that was recommended in knee arthritis and tendinopathies [28]. In this study, the average concentration of platelets in the P-PRP was 790.25 (95%CI, 742.04–838.45) × 10^6^ platelets/mL, which was about three times higher than its concentration in the whole blood and was found to be effective for the treatment of HA. The concentration of growth factors was usually positively related with the platelet’s concentration. P-PRP was obtained by blending the precipitated platelets with PPP in the present study. Comparing the effect of PRP on treating HA, PPP might help demonstrate if there would be several specific components released by platelets that were of biological relevance as well as of potential clinical impact. This will be subject to further experiments and related clinical studies.

Leukocytes in the PRP on one hand had the antibacterial and immunoregulatory abilities and on the other hand could stimulate the expression of cytokines including TNF-α and IL-1β, which further activates the NF-kB signaling pathway by operating the nuclear translocation of p65 and upregulates the downstream catabolic molecules and finally promotes the inflammation [29]. A recent study determined that the concentration of TNF-α and IL-1β in a knee OA model was higher after injection of L-PRP compared to the injection of P-PRP [5]. In another study focusing on the role of L-PRP and P-PRP in the differentiation potential of nucleus pulposus-derived mesenchymal stem cells, researchers claimed that both L-PRP and P-PRP could induce the differentiation of NPMSCs towards the mature NP-like cells, but P-PRP had a much less effect on the activation of the NF-κB signaling pathway [30]. However, some other studies suggested that leukocytes in PRP were related to anti-infection, immune regulation, and angiogenesis activities, and high concentration of leukocytes might have no negative effect on PRP therapy. In addition, some subgroups of leukocytes such as M2 macrophages may have a positive effect on inhibition of inflammation and fibrosis with the activation of Th2 immune response [31]. However, L-PRP does not contain M2 macrophages [32]. In general, it is scientifically reasonable that P-PRP has a better therapeutic effect on knee hemorrhagic arthritis than L-PRP does. We thus assume that P-PRP has an intrinsic advantage compared with L-PRP in the aspect of anti-inflammation ability since low concentration of leukocytes may accompany the absence of TNF-α- and IL-1β-related NF-κB signaling pathway [29]. In this study, we validated the therapeutic effect of P-PRP in the rabbit knee HA model, especially at mild HA.

A clinical research study conducted by Spakova et al. demonstrated that autologous PRP had better effect in comparison with hyaluronic acid in treating knee OA at the early stages [1]. In the present study, the treatment effect of P-PRP was evidently better in the mild HA period of synovitis. Compared to the untreated mild HA group, the group treated with P-PRP had much less cartilage damage and matrix loss. However, for the severe groups, synovitis and cartilage matrix loss present no significant difference between the treated group and untreated group, though inflammation in the treated group was less severe. Evidently, P-PRP was proved to be effective in preventing inflammation and cartilage damage [5]. Nonetheless, the treatment effect of P-PRP for HA at different stages may be different or even the opposite. Collectively, PRP was a promising non-operative treatment for arthritis and Bennell et al. claimed the therapeutic effect of PRP for OA is evidently better than HA in the short term (up to 12 months) [4]. However, several studies have shown that PRP injection may promote the formation of fibrosis [33]. It is a fact that PRP can increase the proliferation and migration ability of fibroblasts [34, 35]. In a recent rat study, though the difference was not remarkable, the PRP group demonstrated a higher percentage of fibrosis than the control group in a gastrocnemius muscle injury model [36]. The consequence of fibrosis may relate with TGF-β1, which has long been considered as a factor in fibrosis. In a recent study, it demonstrated that customized PRP with TGF-β1 neutralization antibody could promote muscle regeneration without increasing fibrosis by using a rat skeletal muscle damage model [37]. In this study, due to the role of TGF-β1, the cartilage fibrosis was inevitable in the late period of synovitis along with the cartilage matrix loss, though the leukocyte concentration was rather low. However, the inflammation in the mild treated group was distinctly less severe than in the untreated group. Therefore, we could reach the conclusion that the therapeutic effect of P-PRP was more notable in mild hemorrhagic arthritis.

There are some limitations in this study. First, this study only chose the time points of 8 and 16 weeks for investigation, and the therapeutic effect of P-PRP in other time points remains unclear. Future studies should include more time points to figure out the best time point of P-PRP treatment. Second, we observed several changes in behavior for rabbits with severe untreated HA knees, which was not timely recorded by video or photograph. In the future, we will conduct related records for animal behaviors or even involving animal gait analysis. Third, this study did not focus on the NF-kB signaling pathway. Immunohistochemistry of specific antibodies and mRNA expression in this pathway should be investigated in the future study. In the histology assessment, this study separated synovium from cartilage because the embedding box was not large enough to contain the whole knee joint of a rabbit. It is definitely preferable to have a precise section of the knee joint in the future study. Though our study suggested P-PRP injection is beneficial for hemorrhagic arthritis, whether the results are of clinical importance needs more trials on the patients.

## Conclusions

In summary, P-PRP presents an effective non-operative treatment for hemorrhagic arthritis, especially at the mild HA. Through our study, we suggest that P-PRP should be applied in the initiation of inflammation to delay or reverse the synovitis and avoid the risk of developing into arthritis. Additionally, as a pre-treatment, P-PRP therapy may decrease the expense and the number of patients finally stepped into TKA surgery. Further clinical studies with specific analysis are needed to give fresh impetus to PRP application.

## Data Availability

We confirm that all relevant data are available from the authors.

## Abbreviations

HA: Hemorrhagic arthritis
TKA: Total knee arthroplasty
PRP: Platelet-rich plasma
P-PRP: Pure PRP
PDGF: Platelet-derived growth factors
TGFβ1: Transforming growth factors β1
IGF: Insulin-like growth factors
PF-4: Platelet factor 4
FGF-2: Fibroblast growth factor 2
VEGF: Vascular endothelial growth factor
L-PRP: PRP with leukocytes
H&E: Hematoxylin and eosin
SD: Standard deviations
95%CI: 95% confidence interval
ECM: Extracellular matrix
GFs: Growth factors
CTGF: Connective tissue growth factor
EGF: Epidermal growth factor
HGF: Hepatocyte growth factor
